# Estimating nationwide cases of sexually transmitted diseases in 2015 from sentinel surveillance data in Japan

**DOI:** 10.1186/s12879-020-4801-x

**Published:** 2020-01-28

**Authors:** Miyuki Kawado, Shuji Hashimoto, Akiko Ohta, Mari S. Oba, Ritei Uehara, Kiyosu Taniguchi, Tomimasa Sunagawa, Masaki Nagai, Yoshitaka Murakami

**Affiliations:** 1 Department of Hygiene, Fujita Health University School of Medicine, 1-98, Kutsukake-cho, Toyoake, Aichi 470-1192 Japan; 2 Division of Public Health, Department of Social Medicine, Saitama Medical University Faculty of Medicine, Moroyama, Saitama, Japan; 3 Department of Medical Statistics, Toho University, Tokyo, Japan; 4 Department of Epidemiology for Community Health and Medicine, Kyoto Prefectural University of Medicine, Kyoto, Japan; 5 Department of Clinical Research, National Mie Hospital, Tsu, Mie Japan; 6 Infectious Disease Surveillance Center, National Institute of Infectious Diseases, Tokyo, Japan; 7 Saitama Medical University, Moroyama, Saitama, Japan

**Keywords:** Surveillance, Sexually transmitted disease, Estimation, Epidemiology

## Abstract

**Background:**

The rates of newly diagnosed cases of sexually transmitted diseases, including genital chlamydial infection and gonorrhea, are important for prevention and control of these diseases. However, nationwide rates are not reported in Japan.

**Methods:**

We used the number of cases of sexually transmitted diseases reported by nationwide sentinel surveillance in 2015, together with the number of all disease outpatients in September 2014 at all medical institutions, drawn from the Survey of Medical Institutions of Japan. The number of cases of sexually transmitted diseases in the total population was estimated using the ratio estimation method with the number of all disease outpatients as auxiliary information. This method is currently used for estimating influenza cases from sentinel surveillance data in Japan.

**Results:**

The estimated number of newly diagnosed cases per 100,000 population in 2015 in Japan was 244 (95% confidence interval 211–277) for genital chlamydial infection, 87 (95% confidence interval 74–100) for genital herpes, 61 (95% confidence interval 29–93) for condyloma acuminatum, and 89 (95% confidence interval 64–113) for gonorrhea.

**Conclusion:**

We estimated the nationwide number of newly diagnosed cases of sexually transmitted diseases in Japan from sentinel surveillance data. This provides useful information for public health policy-making.

## Background

Surveillance is essential for prevention and control of sexually transmitted diseases (STDs) [1]. STD surveillance systems have been established worldwide, and provide information on temporal trends and incidence [2–4]. The rates of new diagnoses of STDs including genital chlamydial infection and gonorrhea in the total population have been shown in many countries. This information indicates the magnitude of the burden of STDs and plays an important role in the planning and evaluation of countermeasures [1, 2, 5]. Exploring the sex and age distribution of STDs could help to clarify high-risk groups, the appropriate targets for countermeasures, and the suitable scale for these countermeasures. The annual comparison of this information could reveal the effect size of countermeasures and suggest the importance of the improvements achieved [1, 6].

In Japan, sentinel surveillance is carried out for influenza, some pediatric diseases and STDs as a part of the National Epidemiological Surveillance for Infectious Diseases (NESID) program [7, 8]. Sentinel medical institutions (SMIs) are selected using well-designed criteria set out in guidelines for sentinel surveillance [8, 9]. The target diseases for STD sentinel surveillance are genital chlamydial infection, genital herpes, condyloma acuminatum and gonorrhea. Reports of recent trends from sentinel surveillance data suggest that the number of males newly diagnosed with condyloma acuminatum has slightly increased, the number of females newly diagnosed with genial chlamydial infection and condyloma acuminatum has slightly decreased, and the numbers newly diagnosed with other STDs were nearly constant [10, 11]. The numbers of cases newly diagnosed with influenza and pediatric diseases in the total population have been estimated using sentinel surveillance data, but the new cases of STDs have not yet been estimated [8, 12, 13]. We aimed to estimate the rates of newly diagnosed STD cases in the total population from sentinel surveillance data in Japan.

## Methods

### Sentinel surveillance for STDs in Japan

Details of NESID in Japan have been described elsewhere [7, 8]. This system is maintained by the Ministry of Health, Labour and Welfare, and uses two ways of collecting information: surveillance for national notifiable diseases and sentinel surveillance. Syphilis is a national notifiable disease, and genial chlamydial infection, genital herpes, condyloma acuminatum and gonorrhea are included in the sentinel system for STDs [8, 10].

Under the sentinel surveillance for STDs, prefectural governments select SMIs following Ministry guidelines [8, 9]. The number of SMIs in the areas covered by each health center is proportional to the population size, increasing by one SMI for every 130,000 population. There are around 900 SMIs, ranging from five to 60 across the 47 prefectures, and the prefectural figures are largely the numbers required by the guidelines [14]. The Ministry guideline recommends selecting SMIs using random sampling from among medical institutions with obstetrics and/or gynecology departments, and those with urology, STD and dermatology departments [8, 9]. In practice, however, SMIs are more likely to be selected on a voluntary basis [14, 15]. Medical institutions are classified as either hospitals if they have 20 or more beds, or clinics if they have fewer than 20 beds. Each SMI reports the monthly number of newly diagnosed STD cases to the health center. Health centers notify the prefectural government and the Ministry through an online reporting platform.

The NESID in Japan uses established definitions of STDs [10, 16]. For genital chlamydial infection and gonorrhea, patients who show possible symptoms are required to meet laboratory case definitions including isolation of pathogens, detection of antigen, genome, and significant rise of specific antibody for *C. trachomatis* and *Neisseria gonorrheae*. For genital herpes and condyloma acuminatum, patients only have to show clinical symptoms; the characteristic herpetiform vesicular or ulcerative lesions, and papillary or cauliflower-like surface warts in anogenital areas.

### Data

We analyzed reports of STD cases in 2015 from SMIs with obstetrics, gynecology, urology and STD departments. The information came from the NESID in collaboration with the National Institute of Infectious Diseases of Japan, which routinely summarizes surveillance data [17]. These reports include the sex- and age-specific number of newly diagnosed cases for four STDs. There are very few SMIs with dermatology departments and the sampling rate is very low, so we did not include data from these in our analysis [14].

To estimate the number of new cases of STDs in Japan, we used the number of all disease outpatients in September 2014 from each hospital obstetrics, gynecology, urology and STD department, and from each clinic, reported in the Survey of Medical Institutions of Japan, with permission from the Ministry [18, 19]. This survey is conducted every three years to determine the status of medical institutions (including all disease outpatients, in September) in Japan.

The data of about 93% of SMIs were linked with the number of all disease outpatients by the name and address of the medical institution. These data included 20,735 cases of genital chlamydial infection, 7021 cases of genital herpes, 4465 cases of condyloma acuminatum and 6808 cases of gonorrhea. They were used to estimate the national numbers of STD cases.

### Estimation of STD cases

The sex- and age-specific numbers of newly diagnosed STD cases in the total population were estimated by the ratio estimation method, with the number of all disease outpatients as auxiliary information. This is the method currently used for estimating influenza cases in the NESID in Japan [12, 13]. The number of STD cases in each prefecture and type of medical institution was estimated as the number of STD cases at SMIs divided by the ratio of the sum of the number of all disease outpatients in SMIs to the sum in all medical institutions. The number of STD cases in the total population was estimated as the total of the STD case estimates for all prefectures and types of medical institution. The four types of medical institution were: 1) hospital obstetrics and gynecology departments, 2) clinic with obstetrics and/or gynecology departments, 3) hospital urology and STD departments, and 4) clinic with urology and/or STD departments. The Additional file 1: Appendix describes the method in detail.

## Results

Table 1 shows the mean number of all disease outpatients in September 2014 at all medical institutions and SMIs. There were 833 SMIs in total (6.7% of all medical institutions). The mean numbers of all disease outpatients were higher in SMIs than all medical institutions. The ratios of mean numbers of all disease outpatients ranged from 1.2 to 1.9 at the four types of medical institution.

**Table 1:** Mean number of all disease outpatients in September 2014 at all and sentinel medical institutions

	Type of medical institution				
	Hospital obstetrics and gynecology departments	Clinic with obstetrics and/or gynecology departments	Hospital urology and STD departments	Clinic with urology and/or STD departments	Total
All medical institutions					
No.	1861	4622	2619	3253	12,355
Mean number of all disease outpatients in September 2014	944	1187	659	1243	1053
Sentinel medical institutions (SMIs)					
No.	170	288	153	222	833
(%)^a^	(9.1)	(6.2)	(5.8)	(6.8)	(6.7)
Mean number of all disease outpatients in September 2014	1549	1486	1234	1451	1443
Ratio^b^	1.6	1.3	1.9	1.2	1.4

*STD* sexually transmitted disease

^a^Proportion of SMIs to all medical institutions

^b^Ratio of mean number of all disease outpatients in SMIs and all medical institutions

Table 2 shows the estimated numbers and rates of newly diagnosed cases of genital chlamydial infection from sentinel surveillance data in 2015 in Japan. The rate per 100,000 population was 244 (95% confidence interval [CI] 211–277). The rate was lower in males than females (male-female ratio of 0.8). In male patients, the highest age-specific rate was in those aged 20–24 years (790). The rate decreased to less than 500 in those aged 35 and older. In female patients, the age-specific rate was over 1000 in those aged 20–29 years, and decreased among those aged 35 and older.

**Table 2:** Estimated numbers and rates of newly diagnosed cases of genital chlamydial infection from sentinel surveillance data in 2015 in Japan

Age (years)	Males				Females				Total				Male-female ratio of rate estimates
	Number (thousands)	Rate (per 100,000 population)			Number (thousands)	Rate (per 100,000 population)			Number (thousands)	Rate (per 100,000 population)			
		Estimate	95% CI			Estimate	95% CI			Estimate	95% CI		
0–14	0.0	0.1	0.0	0.4	0.4	4.7	1.5	7.9	0.4	2.3	0.8	3.9	0.0
15–19	6.2	199.0	167.7	230.3	23.6	802.4	695.8	908.9	29.8	492.2	436.8	547.6	0.2
20–24	24.6	789.5	646.8	932.1	56.4	1899.1	1602.4	2195.8	81.0	1330.3	1155.1	1505.5	0.4
25–29	25.9	778.5	624.7	932.3	40.0	1251.5	1023.2	1479.9	66.0	1010.2	865.9	1154.4	0.6
30–34	24.7	657.2	534.7	779.7	23.8	652.9	516.1	789.7	48.4	655.1	558.1	752.0	1.0
35–39	18.5	433.8	337.7	529.9	14.6	351.1	250.0	452.3	33.1	393.1	321.1	465.0	1.2
40–44	14.3	285.8	218.4	353.3	8.4	172.8	119.2	226.3	22.7	230.0	183.9	276.2	1.7
45–49	9.2	208.3	152.9	263.8	4.5	102.9	35.5	170.2	13.7	156.1	108.0	204.1	2.0
50–54	5.1	126.5	90.3	162.7	2.2	54.6	12.7	96.5	7.3	90.7	62.9	118.6	2.3
55 and older	6.0	26.6	18.6	34.6	1.3	4.8	2.0	7.6	7.3	14.7	10.6	18.7	5.5
Total	134.6	217.6	177.9	257.3	175.1	268.4	221.5	315.2	309.7	243.7	210.5	276.8	0.8

Table 3 shows the estimated numbers and rates of newly diagnosed cases of genital herpes in 2015 in Japan. The rate per 100,000 population was 87 (95% CI, 74–100), and the rate was lower in males than females (male-female ratio of 0.5). In male patients, the highest rate was in those aged 25–29 years (154) and decreased to less than 100 in those aged 40 and older. In female patients, the rate was over 200 in those aged 20–34 years, and decreased in those aged 45 and older.

**Table 3:** Estimated numbers and rates of newly diagnosed cases of genital herpes from sentinel surveillance data in 2015 in Japan

Age (years)	Males				Females				Total				Male-female ratio of rate estimates
	Number (thousands)	Rate (per 100,000 population)			Number (thousands)	Rate (per 100,000 population)			Number (thousands)	Rate (per 100,000 population)			
		Estimate	95% CI			Estimate	95% CI			Estimate	95% CI		
0–14	0.1	0.7	0.0	1.4	0.2	2.4	0.5	4.3	0.2	1.5	0.5	2.6	0.3
15–19	0.8	25.0	15.9	34.2	2.8	96.6	76.7	116.5	3.6	59.8	49.0	70.7	0.3
20–24	3.6	116.8	57.0	176.5	11.4	382.6	317.5	447.7	15.0	246.3	192.4	300.2	0.3
25–29	5.1	153.8	106.9	200.8	12.4	386.6	319.9	453.4	17.5	267.8	224.0	311.7	0.4
30–34	5.2	137.8	85.2	190.3	10.1	276.4	226.8	326.0	15.2	206.1	165.4	246.8	0.5
35–39	5.5	128.2	84.7	171.7	8.2	197.9	162.1	233.7	13.7	162.5	131.4	193.7	0.6
40–44	4.7	94.7	61.4	128.0	6.5	134.2	110.0	158.4	11.2	114.2	93.0	135.4	0.7
45–49	4.2	95.5	62.3	128.7	4.8	111.0	86.9	135.1	9.0	103.2	80.8	125.6	0.9
50–54	2.8	69.7	46.5	92.9	4.2	104.4	82.5	126.3	7.0	87.0	70.5	103.5	0.7
55 and older	5.5	24.2	17.4	31.0	12.2	44.8	34.7	54.8	17.7	35.4	29.2	41.7	0.5
Total	37.5	60.6	43.4	77.8	72.8	111.6	95.9	127.2	110.3	86.8	73.8	99.8	0.5

Table 4 shows the estimated numbers and rates of newly diagnosed cases of condyloma acuminatum in 2015 in Japan. The rate per 100,000 population was 61 (95% CI, 29–93), and the rate was higher in males than females (male-female ratio of 1.5). In male patients, the highest rate was in those aged 25–29 years (226) and decreased to less than 200 in those aged 35 and older. In female patients, the rate was over 300 in those aged 20–24 years, and decreased after age 30.

**Table 4:** Estimated numbers and rates of newly diagnosed cases of condyloma acuminatum from sentinel surveillance data in 2015 in Japan

Age (years)	Males				Females				Total				Male-female ratio of rate estimates
	Number (thousands)	Rate (per 100,000 population)			Number (thousands)	Rate (per 100,000 population)			Number (thousands)	Rate (per 100,000 population)			
		Estimate	95% CI			Estimate	95% CI			Estimate	95% CI		
0–14	0.0	0.6	0.0	1.3	0.0	0.1	0.0	0.7	0.1	0.3	0.0	0.8	6.4
15–19	0.7	21.2	11.2	31.3	2.2	75.0	54.1	95.9	2.9	47.4	34.2	60.5	0.3
20–24	5.7	183.5	50.3	316.7	9.2	309.5	192.8	426.2	14.9	244.9	124.3	365.5	0.6
25–29	7.5	226.0	51.1	400.8	7.1	222.6	150.9	294.3	14.7	224.3	105.8	342.8	1.0
30–34	8.2	219.9	41.0	398.8	4.8	132.0	77.6	186.3	13.1	176.6	62.1	291.0	1.7
35–39	6.3	147.9	26.7	269.1	3.3	79.2	44.9	113.5	9.6	114.0	39.4	188.7	1.9
40–44	6.3	126.2	41.8	210.5	2.5	52.1	32.3	71.8	8.8	89.6	40.3	138.9	2.4
45–49	3.5	79.1	38.5	119.8	1.2	27.7	13.5	41.9	4.7	53.6	27.6	79.6	2.9
50–54	2.7	67.0	24.1	109.8	0.6	15.5	8.5	22.5	3.3	41.3	17.7	65.0	4.3
55 and older	4.5	19.7	9.9	29.5	1.1	4.1	1.7	6.5	5.6	11.2	5.7	16.6	4.8
Total	45.5	73.6	23.3	123.8	32.1	49.2	32.6	65.8	77.6	61.0	29.0	93.1	1.5

Table 5 shows the estimated numbers and rates of newly diagnosed cases of gonorrhea in 2015 in Japan. The rate per 100,000 population was 89 (95% CI, 64–113), and the rate was higher in males than females (male-female ratio of 2.6). In male patients, the highest rate was in those aged 20–24 years (540) and decreased to less than 200 in those aged 40 and older. In female patients, the rate was over 200 in those aged 20–29 years, and decreased after age 30.

**Table 5:** Estimated numbers and rates of newly diagnosed cases of gonorrhea from sentinel surveillance data in 2015 in Japan

Age (years)	Males				Females				Total				Male-female ratio of rate estimates
	Number (thousands)	Rate (per 100,000 population)			Number (thousands)	Rate (per 100,000 population)			Number (thousands)	Rate (per 100,000 population)			
		Estimate	95% CI			Estimate	95% CI			Estimate	95% CI		
0–14	0.0	0.1	0.0	0.6	0.1	1.5	0.0	3.0	0.1	0.8	0.0	1.6	0.1
15–19	4.8	153.5	124.3	182.8	3.4	114.6	89.6	139.5	8.2	134.6	113.9	155.4	1.3
20–24	16.9	540.4	407.3	673.5	8.6	289.6	158.3	420.9	25.5	418.1	315.4	520.9	1.9
25–29	15.5	464.0	347.8	580.3	7.1	222.5	56.5	388.5	22.6	345.7	236.5	455.0	2.1
30–34	12.7	337.5	247.9	427.1	4.0	109.8	0.0	221.5	16.7	225.3	146.2	304.3	3.1
35–39	9.7	226.6	172.9	280.3	3.4	81.2	0.0	180.3	13.0	154.9	95.6	214.3	2.8
40–44	8.2	165.2	128.0	202.5	2.9	59.4	0.0	120.7	11.1	113.0	75.2	150.8	2.8
45–49	5.8	131.9	93.5	170.3	1.5	33.9	0.0	77.3	7.3	83.4	52.1	114.6	3.9
50–54	3.2	80.2	58.1	102.4	1.1	27.1	2.2	52.1	4.3	53.8	36.3	71.3	3.0
55 and older	3.4	15.2	11.8	18.5	0.4	1.5	0.7	2.3	3.8	7.7	6.1	9.3	10.3
Total	80.2	129.7	101.4	158.0	32.4	49.7	14.5	84.9	112.6	88.6	63.9	113.3	2.6

## Discussion

Our study investigated the nationwide incidence of STDs in Japan using sentinel surveillance data for infectious diseases. This is the first study of which we are aware to estimate the rates of new diagnoses of STDs from sentinel surveillance data in Japan [10, 11, 20]. However, the coverage of SMIs among the national population is used in several other countries to estimate these numbers [21, 22]. In Japan, the population coverage of each medical institution is not known because of the free access healthcare system [15, 20]. Previous studies have found that if SMIs were recruited on a voluntary basis for influenza sentinel surveillance, medical institutions with more patients with influenza would usually be selected as SMIs [12–14]. An assumption that SMIs were randomly selected would therefore lead to overestimation of influenza incidence. Using the number of all disease outpatients as auxiliary information avoids this assumption [13, 15]. A previous study showed that this method would lead to relatively accurate estimates of influenza incidence using influenza sentinel surveillance data from SMIs [13]. We found that the mean numbers of all disease outpatients were higher in SMIs than all medical institutions (Table 1), suggesting that medical institutions with more patients with STDs were usually selected as SMIs in the STD sentinel surveillance. The method with the number of all disease outpatients as auxiliary information was expected to reduce the bias of estimates of the number of newly diagnosed cases of STDs in the total population from non-random selection of SMIs. Further study on the validity of this method of estimating the numbers of STD cases is needed [13, 15].

In Japan, countermeasures for STD prevention and control are being implemented on the basis of the Ministry of Health, Labor and Welfare’s Guideline for Preventing Specific Infectious Diseases Regarding Sexually Transmitted Diseases [23]. The guideline includes improvements seeking to understand the occurrence and trends of STDs, disseminate knowledge and educate people, and provide opportunities for screening and consultation. The NESID is fundamental for grasping the occurrence and trends of STDs in Japan. The estimation of rates of newly diagnosed STD cases in the present study is precisely the kind of improvement called for to understand the occurrence and trends of STDs. If this estimation method was embedded in the NESID system, estimates of STD cases in Japan could be shown on an annual basis, providing more information [17]. In the previous studies, genital chlamydial infection has been identified as the most common STD in Japan, and it has been found to be especially common among young women [10, 11]. In the screening program for genital chlamydial infection, free and anonymous voluntary counseling and testing are carried out nationwide in public health centers [24]. Our rate estimates not only confirmed these previous findings of genital chlamydial infection, but also indicated the magnitude of the burdens and suggested the importance of countermeasures against the disease. Observing rate estimates for genital chlamydial infection, genital herpes, condyloma acuminatum and gonorrhea cases annually would certainly be useful for the planning and evaluation of these countermeasures, but concrete action for the improvement of such interventions would require further information [1, 6].

The estimated rates of newly diagnosed cases of the four examined STDs in 2015 in Japan enables comparisons of the magnitude of the burden of STDs between Japan and other countries, including the United States and European countries [2–4]. The results of these comparisons may be useful for public health policy-making [23]. We found 244 newly diagnosed genital chlamydial infection cases in 2015 in Japan per 100,000 population, and a male-female ratio of 0.8. The surveillance report in 2015 in the United States found 475 new cases per 100,000 population and a male-female ratio of 0.5 [3]. In Europe, the rate and male-female ratio varied widely among countries, but the overall rate and ratio were 175 and 0.7 [25]. The WHO report in 2018 included no rates for genital chlamydial infection cases in other countries [2]. The rate in Japan was between that found in the United States and the average level in Europe, and the male-female ratio in Japan was higher than both that found in the United States and the average level in Europe. Interpreting these results is not easy. There may be several reasons for these differences, including the extent of access to sensitive diagnostics, differences in surveillance data collection, variations in national testing policies, the level of testing policy implementation, and actual differences in incidence rates [3, 25, 26].

We found 89 newly diagnosed cases of gonorrhea per 100,000 population, and a male-female ratio of 2.6. The United States surveillance report in 2015 found a rate of 123 and a male-female ratio of 1.3 [3]. In Europe, there were again considerable variations among countries, but the overall rate and ratio were 19 and 3.3 [27]. The rate and male-female ratio in Japan were between those found in the United States and the average level in Europe. These differences in rates and male-female ratios between the United States, European countries and Japan might be linked to real differences as well as differences in healthcare systems, access to services, and surveillance data collection [3, 26, 27]. The WHO report showed that the rate in males aged 15–49 years in 2016–2017 varied between 0.0 and 387.5 per 100,000 population across 64 countries [2]. This range may be associated with the large differences mentioned before.

We found 61 newly diagnosed cases of condyloma acuminatum per 100,000 population, and a male-female ratio of 1.5. The rates of this disease were not included in the surveillance reports in the United States and Europe or from the WHO [2–4]. A systematic review, however, showed that before 2010, the rates in seven countries ranged from 118 in Spain to 205 in the United States [28]. Vaccination against human papillomavirus, which causes condyloma acuminatum, was introduced in the United States in 2006, in most European countries in 2007–2010, and in Japan in 2013 [20, 29, 30]. High vaccination coverage is expected to lead to great decrease in the rate of condyloma acuminatum [30]. Observing future trends in the rate in Japan would be important.

We found 87 new cases of genital herpes per 100,000 population, and a male-female ratio of 0.5. Again, the rates for this disease were not included in surveillance reports from the United States, Europe or the WHO [2–4]. However, national surveillance reports showed rates per 100,000 population of 62 in England and 120 in the Netherlands [31, 32]. Genital herpes infections are subclinical. Seroprevalence of herpes simplex virus has been surveyed in the United States and several European countries [33].

This study had some limitations. We estimated the rates of newly diagnosed cases of four STDs in 2015 in Japan. Other common STDs, such as chancroid and trichomonas vaginalis, were not included [7, 8]. Syphilis was also not included in our estimation, because its reporting is mandatory in Japan, so no estimate of its incidence is needed. STDs are often asymptomatic and may not be diagnosed [1, 2]. The estimated rates might therefore underestimate the actual incidence of infections [3, 5]. Information on the magnitude of underestimation of the four STDs in Japan is unknown, and will be an important target for future studies. The report data might include some cases which did not meet the definition of STDs [16]. We used the data for about 93% of SMIs linked with the number of all disease outpatients, and this may have decreased the accuracy of our estimates. We were able to make a broad comparison of the rates of newly diagnosed cases of four STDs between Japan and other countries including the United States and European countries [2–4]. Better comparison would require more advanced analysis.

## Conclusion

We estimated the nationwide rates of newly diagnosed STD cases of 2015 in Japan from sentinel surveillance data. The rates per 100,000 population were 244 for genital chlamydial infection, 87 for genital herpes, 61 for condyloma acuminatum, and 89 for gonorrhea. It was suggested that the rates of genital chlamydial infection and gonorrhea were between those found in the United States and the average level in Europe using their surveillance data. Our estimates provide useful information for public health policy-making.

## Supplementary information

**Additional file 1:** Appendix. Method of estimating STD cases.

## Data Availability

The datasets analyzed in this study are not available because we collected data of STD reports in collaboration with the National Institute of Infectious Diseases of Japan and data of all disease outpatients with permission from the Ministry of Health, Labour and Welfare of Japan.

## Abbreviations

CI: Confidence interval
NESID: National Epidemiological Surveillance for Infectious Diseases
SMI: Sentinel medical institution
STD: Sexually transmitted disease

## References

[1] Zenilman JM, Nelson KE, Williams CM, Graham NMH (2001). Sexually transmitted diseases. Infectious disease epidemiology: theory and practice.

[2] World Health Organization. Report on global sexually transmitted infection surveillance. 2018. https://apps.who.int/iris/bitstream/handle/10665/277258/9789241565691-eng.pdf?ua=1. Accessed 18 November 2019.

[3] Centers for Disease Control and Prevention, US. Sexually Transmitted Disease Surveillance. 2017. https://www.cdc.gov/std/stats17/2017-STD-Surveillance-Report_CDC-clearance-9.10.18.pdf. Accessed 18 November 2019.

[4] European Centre for Disease Prevention and Control. Annual epidemiological report 2014—sexually transmitted infections, including HIV and blood-borne viruses. 2015. https://ecdc.europa.eu/sites/portal/files/media/en/publications/Publications/sexually-transmited-infections-HIV-AIDS-blood-borne-annual-epi-report-2014.pdf. Accessed 18 November 2019.

[5] Chen MY, Tabrizi SN (2015). Challenges to the management of curable sexually transmitted infections. BMC Infect Dis.

[6] Cates W, Holmen KK, Last JM, Wallace RB (1992). Sexually transmitted diseases. Maxcy-Rosenau-Last's public health and preventive medicine, 13th editon.

[7] Taniguchi K, Hashimoto S, Kawado M, Murakami Y, Izumida M, Ohta A (2007). Overview of infectious disease surveillance system in Japan, 1999-2005. J Epidemiol.

[8] Infectious Disease Surveillance Center, National Institute of Infectious Diseases. The National Epidemiological Surveillance of Infectious Diseases in Compliance with the Enforcement of the New Infectious Diseases Control Law. IASR (Infectious Agents Surveillance Report). 1999;20(4):230. http://idsc.nih.go.jp/iasr/20/230/de2309.html. Accessed 18 Nov 2019.

[9] Tuberculosis and Infectious Diseases Control Division, Health Service Bureau, Ministry of Health, Labour and Welfare of Japan. The Guideline for National Epidemiological Surveillance of Infectious Diseases (kansensho-hasseidokochosajigyo-jissiyoko). Weekly News of Public Health (Shukan-hoken-eisei). 1999;998:14–26 (in Japanese).

[10] Infectious Disease Surveillance Center, National Institute of Infectious Diseases. Sexually transmitted diseases in Japan as of 2007. IASR (Infectious Agents Surveillance Report). 2008;29(9):343. http://idsc.nih.go.jp/iasr/29/343/tpc343.html. .

[11] Arakawa S, Arima Y, Takahashi T, Tanihata T, Sunagawa T (2016). Recent trends of sexually transmitted diseases. Rinsho-to-kenkyu.

[12] Kawado M, Hashimoto S, Murakami Y, Izumida M, Ohta A, Tada Y (2007). Annual and weekly incidence rates of influenza and pediatric diseases estimated from infectious disease surveillance data in Japan, 2002–2005. J Epidemiol.

[13] Kawado M, Hashimoto S, Ohta A, Oba MS, Taniguchi K, Sunagawa T (2018). Improvement of influenza incidence estimation using auxiliary information in sentinel surveillance in Japan. Open Infect Dis J.

[14] Murakami Y, Hashimoto S, Taniguchi K, Kosaka K, Fuchigami H, Nagai M (2003). Distribution of monitoring stations in the surveillance of infectious disease after the legislation of new infectious disease control law in Japan. Nihon Koshu Eisei Zasshi..

[15] Hashimoto S, Murakami Y, Taniguchi K, Shindo N, Osaka K, Fuchigami H (2003). Annual incidence rate of infectious diseases estimated from sentinel surveillance data in Japan. J Epidemiol.

[16] Ministry of Health, Labour and Welfare of Japan. Request of Report by Doctor Based on the Infectious Diseases Control Law. 2019. https://www.mhlw.go.jp/stf/seisakunitsuite/bunya/kenkou_iryou/kenkou/kekkaku-kansenshou/kekkaku-kansenshou11/01.html. Accessed 18 Nov 2019 (in Japanese).

[17] National Institute of Infectious Diseases, Japan. Infectious Diseases Weekly Report. 2019. https://www.niid.go.jp/niid/en/idwr-e.html. Accessed 18 Nov 2019.

[18] Ministry of Health, Labour and Welfare of Japan. List of Statistical Surveys conducted by Ministry of Health, Labour and Welfare. 2019. https://www.mhlw.go.jp/toukei/itiran/eiyaku.html. Accessed 18 Nov 2019.

[19] Statistics and Information Department, Minister's Secretariat, Ministry of Health, Labour and Welfare of Japan. The Survey of Medical Institutions 2014. Tokyo: Health and Welfare Statistics Association of Japan; 2016 (in Japanese).

[20] Takahashi S, Takeyama K, Kunishima Y, Shimizu T, Nishiyama N, Hotta H (2004). Incidence of sexually transmitted diseases in Hokkaido, Japan, 1998 to 2001. Infect Chemother.

[21] Fleming DM, Zambon M, Bartelds AI (2000). Population estimates of persons presenting to general practitioners with influenza-like illness, 1987-96: a study of the demography of influenza-like illness in sentinel practice networks in England and Wales, and in the Netherlands. Epidemiol Infect.

[22] Schlaud M, Brenner MH, Hoopmann M, Schwartz FW (1998). Approaches to the denominator in practice-based epidemiology: a critical overview. J Epidemiol Community Health.

[23] Ministry of Health, Labour and Welfare of Japan. Guideline for Preventing Specific Infectious Diseases Regarding Sexually Transmitted Diseases. 2018. https://www.mhlw.go.jp/file/06-Seisakujouhou-10900000-Kenkoukyoku/0000186685.pdf. Accessed 18 Nov 2019 (in Japanese).

[24] Hatori T, Nakamura T, Tsukui S (2013). The importance of using a test to detect chlamydia trachomatis infection in patients undergoing counseling and testing for sexually transmitted diseases at a public health center. Nihon Koshu Eisei Zasshi.

[25] European Centre for Disease Prevention and Control. Chlamydia infection. In: ECDC. Annual epidemiological report for 2017. 2019. https://ecdc.europa.eu/sites/portal/files/documents/chlamydia-infection-annual-epidemiological-report-2017.pdf. Accessed 18 November 2019.

[26] Götz HM, van Oeffelen LA, Hoebe CJPA, van Benthem BH (2019). Regional differences in chlamydia and gonorrhoeae positivity rate among heterosexual STI clinic visitors in the Netherlands: contribution of client and regional characteristics as assessed by cross-sectional surveillance data. BMJ Open.

[27] European Centre for Disease Prevention and Control. Gonorrhoea. In: ECDC. Annual epidemiological report for 2016. 2018. https://ecdc.europa.eu/sites/portal/files/documents/AER_for_2016-gonorrhoea.pdf. Accessed 18 Nov 2019.

[28] Patel H, Wagner M, Singhal P, Kothari S (2013). Systematic review of the incidence and prevalence of genital warts. BMC Infect Dis.

[29] Flagg EW, Torrone EA (2018). Declines in anogenital warts among age groups most likely to be impacted by human papillomavirus vaccination, United States, 2006–2014. Am J Public Health.

[30] Bonanni P, Levi M, Latham NB, Bechini A, Tiscione E, Lai P (2011). An overview on the implementation of HPV vaccination in Europe. Hum Vaccin.

[31] Public Health England. Sexually transmitted infections and screening for chlamydia in England, 2018. 2019. https://assets.publishing.service.gov.uk/government/uploads/system/uploads/attachment_data/file/806118/hpr1919_stis-ncsp_ann18.pdf#search=%27Public+Health+England.+Sexually+transmitted+infections+and+screening+for+chlamydia+in+England%2C+2017%27. Accessed 18 Nov 2019.

[32] van den Broek IV, Verheij RA, van Dijk CE, Koedijk FD, van der Sande MA, van Bergen JE (2010). Trends in sexually transmitted infections in the Netherlands, combining surveillance data from general practices and sexually transmitted infection centers. BMC Fam Pract.

[33] Glinšek Biškup U, Uršič T, Petrovec M (2015). Laboratory diagnosis and epidemiology of herpes simplex 1 and 2 genital infections. Acta Dermatovenerol Alp Pannonica Adriat.

