# Supplementary material for: Estimating nationwide cases of sexually transmitted diseases in 2015 from sentinel surveillance data in Japan
Source: BMC Infect Dis. 2020 Jan 28;20:77. doi: 10.1186/s12879-020-4801-x (PMC6986098; doi:10.1186/s12879-020-4801-x)
Supplement: Supplementary file 1 — Additional file 1: Appendix. Method of estimating STD cases. [file 12879_2020_4801_MOESM1_ESM.pdf]

Additional file 1

Appendix: Method of estimating STD cases

Estimating nationwide cases of sexually transmitted diseases in 2015 from sentinel surveillance data in Japan.

Miyuki Kawado, Shuji Hashimoto, Akiko Ohta, Mari S. Oba, Ritei Uehara, Kiyosu Taniguchi, Tomimasa Sunagawa, Masaki Nagai and Yoshitaka Murakami.

Method of estimating STD cases

The method for estimating STD cases was as follows. Let n, ν, and ξ be the number of SMIs, the number of all medical institutions, and the total number of all disease outpatients in all medical institutions. Let Y_i and x_i be STD cases and the number of all disease outpatients in the i-th SMI, for $i = 1, 2, \cdots, n$. Let $\bar{Y}$ and $\bar{x}$ be the mean of $\{Y_i\}$ and $\{x_i\}$.

Let α be the total STD cases in all medical institutions. By using auxiliary information, the estimate of α is given to be $\hat{\alpha} = \xi \times R$, where $R = \bar{Y}/\bar{x}$; i.e., and the total STD cases in all medical institutions is estimated as the total cases in SMIs $(n \times \bar{Y})$ divided by the ratio of the sum of the numbers of all disease outpatients in SMIs to the sum of those in all medical institutions $(n \times \bar{x}/\xi)$. The approximate confidence interval for α is $(\hat{\alpha} - 1.96 \times s\, , \hat{\alpha} + 1.96 \times s)$, where s^2 is an estimate of variance of $\hat{\alpha}$ and is $\nu^2\{\sum(Y_i - R \times x_i)^2/(n-1)\}(1/n - 1/\nu)$.

Consider that the total STD cases in some strata, such as type of medical institution and prefecture, are estimated using the above methods. Let k be the number of strata, $\hat{\alpha}_1$, $\hat{\alpha}_2$, $\cdots$, $\hat{\alpha}_k$ the estimated STD cases in the strata, and ${s_1}^2$, ${s_2}^2$, $\cdots$, ${s_k}^2$ their estimated variances. The approximate confidence interval for the total cases is given as $(\hat{\alpha}_t - 1.96 \times s_t\, , \hat{\alpha}_t + 1.96 \times s_t)$, where $\hat{\alpha}_t = \hat{\alpha}_1 + \hat{\alpha}_2 + \cdots + \hat{\alpha}_k$ and ${s_t}^2 = {s_1}^2 + {s_2}^2 + \cdots + {s_k}^2$.
